# Sex-specific effects of the histone variant H2A.Z on fear memory, stress-enhanced fear learning and hypersensitivity to pain

**DOI:** 10.1038/s41598-020-71229-x

**Published:** 2020-08-31

**Authors:** Firyal Ramzan, Samantha D. Creighton, Meaghan Hall, Jennet Baumbach, Malak Wahdan, Sandra J. Poulson, Vassilia Michailidis, Gilda Stefanelli, Klotilda Narkaj, Cindy S. Tao, Dure Khan, Carl F. D. Steininger, Brandon J. Walters, D. Ashley Monks, Loren J. Martin, Iva B. Zovkic

**Affiliations:** 1 Department of Psychology, University of Toronto Mississauga, 3359 Mississauga Rd. CCT 4071, Mississauga, ON L5L 1C6 Canada; 2 Department of Medicine, Imperial College London, London, SW7 2AZ UK; 3 Department of Cell & Systems Biology, University of Toronto Mississauga, Mississauga, ON L5L, 1C6 Canada; 4 Department of Psychology, University of Toronto Scarborough, Scarborough, ON M1C 1A4 Canada; 5 Department of Biology, University of Toronto Mississauga, Mississauga, ON L5L 1C6 Canada

**Keywords:** Neuroscience, Epigenetics in the nervous system, Epigenetics and behaviour

## Abstract

Emerging evidence suggests that histone variants are novel epigenetic regulators of memory, whereby histone H2A.Z suppresses fear memory. However, it is not clear if altered fear memory can also modify risk for PTSD, and whether these effects differ in males and females. Using conditional-inducible H2A.Z knockout (cKO) mice, we showed that H2A.Z binding is higher in females and that H2A.Z cKO enhanced fear memory only in males. However, H2A.Z cKO improved memory on the non-aversive object-in-place task in both sexes, suggesting that H2A.Z suppresses non-stressful memory irrespective of sex. Given that risk for fear-related disorders, such as PTSD, is biased toward females, we examined whether H2A.Z cKO also has sex-specific effects on fear sensitization in the stress-enhanced fear learning (SEFL) model of PTSD, as well as associated changes in pain sensitivity. We found that H2A.Z cKO reduced stress-induced sensitization of fear learning and pain responses preferentially in female mice, indicating that the effects of H2A.Z depend on sex and the type of task, and are influenced by history of stress. These data suggest that H2A.Z may be a sex-specific epigenetic risk factor for PTSD susceptibility, with implications for developing sex-specific therapeutic interventions.

## Introduction

The capacity to remember fear-related cues is an important survival-promoting adaptation, but it can become maladaptive in some psychiatric conditions, such as post-traumatic stress disorder (PTSD). PTSD develops in response to a traumatic experience and is characterised by intrusive memories of the trauma and sensitization to fear, which presents as enhanced formation of new memories for relatively mild fearful stimuli^1–7^. PTSD disproportionately affects women, who have twice the risk of developing the disorder^8^, and who are more prone to developing new fear memories compared to men with PTSD^9–11^. In rodents, fear sensitization is modeled with stress-enhanced fear learning (SEFL), a paradigm in which exposure to a strong stressor (used to model traumatic experience) results in strengthened acquisition of fear memory compared to mice without prior stress exposure^10^. This sensitization effect is observable within 24 h after stress, is long lasting, and is associated with a range of PTSD-like symptoms, including increased anxiety and impaired fear extinction^10^.

Some evidence suggests that PTSD may also sensitize patients to various forms of painful stimuli. Rates of chronic pain are higher among patients with PTSD compared to the general population, pain onset begins after PTSD symptoms emerge, and intensity of pain positively correlates with the severity of PTSD^12^. Although sex differences in pain sensitivity in PTSD patients have not been widely studied, sex differences in pain sensitivity in the general population are widely reported, with women exhibiting higher prevalence of chronic pain and lower pain thresholds compared to men^13–15^. One recent study in rodents showed that exposure to a single prolonged stressor, often used to induce SEFL, potentiates pain sensitivity to a similar extent in both sexes, but that this sensitization is achieved through distinct molecular mechanisms^16^, suggesting that unique biological risk factors may regulate sensitivity to stress-induced alterations in pain sensitivity.

Indeed, sex differences have been described for the cellular and molecular mechanisms of fear memory, stress, and pain responses. For fear circuitry, differences exist in synaptic function, circuit activity, and in the size and volume of hippocampus and amygdala subregions, even when memory performance is similar (reviewed in^17,18^). Similarly, acute stress exposure results in widely distinct transcriptional responses in males and females^19^ and stress exposure may sensitize pain responses through sex-specific mechanisms. For example, knocking out the nociceptin/orphanin FQ receptor (NOP) protects against stress-potentiated pain sensitivity only in males^16^, indicating that molecular underpinnings of stress-potentiated pain sensitivity may be sex-specific.

Epigenetic modifications emerged as strong candidate mechanisms for understanding stable behavioral adaptations, such as PTSD, because of their potential to persist over long periods of time and their vital role in the maintenance of enduring fear memories^20–23^. Indeed, epigenetic mechanisms are involved in adapting to stressors and are emerging as regulators of pain responses in human patients^24^, making them especially promising as potential links between fear memory, PTSD, and pain. Although the epigenetic basis of fear potentiation in SEFL is not clear, stressors utilized in SEFL paradigms result in extensive epigenetic changes across the genome, including genes with known roles in stress regulation and neural plasticity, such as the glucocorticoid-receptor encoding gene, as well as plasticity-related genes like *Bdnf* and the postsynaptic density gene, *Dlgap2*^25–28^. Moreover, stressful experiences change transcriptional and epigenetic responses to subsequent stressors^29^, suggesting that stress exposure may sensitize fear learning at least in part through epigenetic factors.

Sex differences in the epigenetic basis of fear learning and SEFL have not been widely studied, but there is evidence for sex differences in the binding and expression of various epigenetic markers in the brain^30,31^, which have functional implications for behavior. For example, sex differences in fear extinction have been linked with sex-differences in epigenetic marks in the medial prefrontal cortex^32^ and sex-specific differences in DNA methylation have been linked with risk for PTSD^33^. Together, these data indicate that sex differences in the epigenome may contribute to sex differences in fear memory and associated conditions, including PTSD.

Most epigenetic studies relating to stress and fear memory have focused on DNA methylation and post-translational modifications of histones, but recent studies from our lab and others show that histone variants are a novel epigenetic regulators of memory that have a unique relevance in neurons^22,34–38^. DNA is packaged into the nucleus by wrapping in 147 base pairs segments around an octamer of histone proteins (2 copies of histones H2A, H2B, H3 and H4) to form nucleosomes, the building blocks of chromatin. This packaging creates a physical barrier to transcription that is altered by post-translational modifications of histone proteins, but also by replacing canonical histones with their functionally and structurally distinct variants^39^. In contrast to replication-dependent canonical histones (H2A, H2B, H3, and H4), their non-allelic histone variants are replication-independent, and as such, they become the primary source of histones in post-mitotic neurons^38–40^. Histone variants are of particular interest in studies of behavioral regulation because of their higher potential level of selectivity as therapeutic targets. Specifically, variants represent individual protein targets that have a more sparse distribution compared to DNA methylation and post-translational modifications of histones, which are widely distributed across the genome and various histone types and as such, are much less specific targets. Indeed, H2A.Z represents approximately 10% of total H2A^41^ and tends to be strongly positioned at nucleosomes flanking transcription start sites^34^, which theoretically makes this variant a more precise target for therapeutic intervention. Moreover, histone variants form an important part of the histone code through interactions with other epigenetic modifications, including a well characterized exclusion of H2A.Z from methylated DNA in non-neuronal cells^40,42^, indicating that a comprehensive understanding of epigenetic regulation of neural plasticity necessitates the study of histone variants. In contrast to post-translational modifications, which tend to be short lasting^21,43^, histone H2A.Z incorporation into nucleosomes is stably altered after fear learning^22^, suggesting that histone H2A.Z may also be relevant for regulating stable phenomena, such as PTSD.

H2A.Z is a variant of the canonical histone H2A that acts as a negative regulator of fear memory, whereby virally-mediated reduction of H2A.Z in the hippocampus of male mice enhances associative fear memory^34,35^. However, it is not clear whether this effect holds true in females, or if the effect of H2A.Z on memory is unique to fear memory. It is also not known whether improved memory observed with H2A.Z reduction represents a general improvement in associative learning, or if the loss of H2A.Z could be maladaptive by also potentiating fear-related symptoms in models of PTSD. To address these questions, the goal of the present study is to investigate sex differences in H2A.Z binding and functional differences in fear memory, SEFL, and associated changes in pain sensitivity using an inducible-conditional H2A.Z knockout mouse with selective loss of H2A.Z in CamKIIα-positive cells.

## Results

### H2A.Z binding is higher in female than in male mice

**Figure 1:**
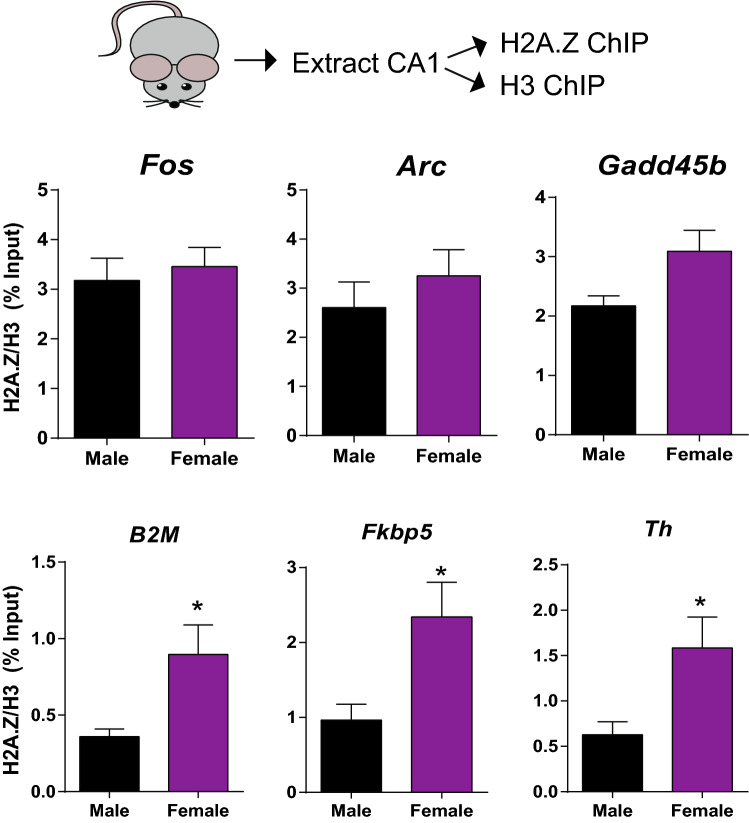
Females have higher H2A.Z binding than males in a gene-specific way. ChIP was conducted to assess binding of H2A.Z, normalized for H3 binding at the same loci, at several genes related to neural plasticity. There were no sex differences in H2A.Z binding at *Fos, Arc,* or *Gadd45b*, but females had significantly more H2A.Z at *B2m, Fkbp5*, and *Th* compared to males. Data are shown as Mean ± SEM, *p ≤ 0.05. N = 8–10/sex.

Previous studies have exclusively quantified H2A.Z binding in the hippocampus of male mice. With the aim of characterizing potential sex differences in H2A.Z occupancy, we utilized chromatin immunoprecipitation (ChIP) in area CA1 to assess H2A.Z binding on proximal promoters of several genes that previously showed evidence of H2A.Z binding in genome-wide studies conducted in the hippocampus^34^. To ensure that any differences in H2A.Z binding were not caused by differences in total nucleosome occupancy in male and female mice, all H2A.Z data were normalized to H3 levels at the same locus. There were no differences in H2A.Z binding on *Fos*, *Arc*, or *Gadd45b*, but females had significantly higher H2A.Z binding on beta-2-microglobulin (*B2m*; t_17_ = 2.56, p = 0.02)*,* FK506 Binding Protein 5 (*Fkbp5*; t_15_ = 2.60, p = 0.02), and Tyrosine Hydroxylase (*Th*; t_17_ = 2.48, p = 0.024), all of which are implicated in neural plasticity and memory^44–46^. There were no sex differences in H3 binding for any of the genes examined (data not shown). These data suggest that H2A.Z exhibits gene-specific occupancy differences in male and female mice, with females having higher H2A.Z binding compared to males (Fig. 1).

#### Conditional-inducible H2A.Z knockout in CaMKIIα-positive neurons

**Figure 2:**
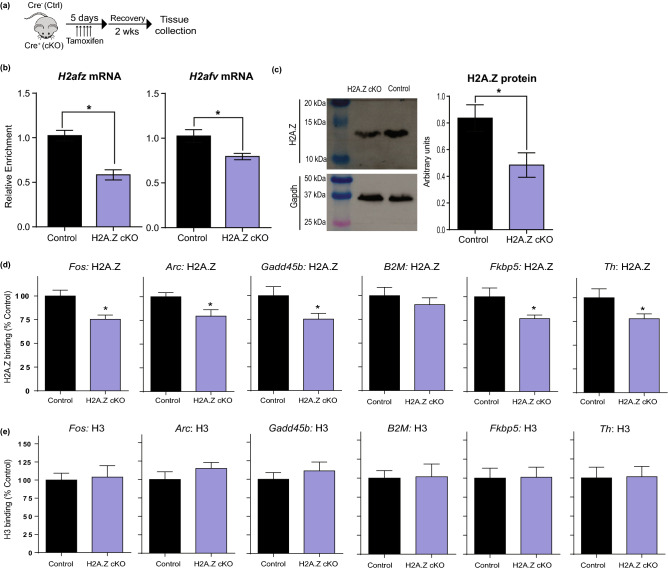
Validation of inducible conditional H2A.Z knockout. (**a**) Design used to induce H2A.Z deletion in CamKIIα-positive neurons in adult mice. All mice were floxed for *H2afz* and *H2afv*. Conditional knockout was induced by crossing floxed mice with *CamKIIα*-*Cre*ER mice, such that *Cre* positive (Cre^+^) mice exhibited H2A.Z deletion upon treatment with tamoxifen, whereas *Cre* negative (Cre^-^) mice treated with tamoxifen served as controls. (**b**) The deletion of each H2A.Z-encoding gene was validated by qPCR at the mRNA level. N = 10–12/group; (**c**) reduced protein was validated using immunoblotting for total H2A.Z in the hippocampus. N = 6/group; (**d**) reduced H2A.Z binding was validated using H2A.Z ChIP; (**e**) Preservation of nucleosomes was validated using histone H3 ChIP N = 6/group. Data are shown as Mean ± SEM, *p ≤ 0.05.

Initial studies on the function of H2A.Z only targeted one of the two H2A.Z-encoding genes (*H2afz,* encodes H2A.Z1), leaving the possibility that a compensatory effect of *H2afv* (encodes H2A.Z2) is responsible for improved memory in *H2afz*-depleted mice. To address this possibility, we utilized a conditional-inducible H2A.Z knockout (H2A.Z cKO) model, in which both H2A.Z-encoding genes were selectively deleted in neurons that express CaMKIIα. The H2A.Z knockout was induced by tamoxifen injections in adult mice delivered ~ 2 weeks before testing. The depletion of each individual H2A.Z-encoding gene, *H2afz* (t_20_ = 5.32, *p* < 0.001) and *H2afv* (t_20_ = 2.64, *p* = 0.02), was confirmed using qPCR and the reduction in total H2A.Z protein was confirmed with immunoblotting (t_10_ = 2.59, *p* = 0.03; Fig. 2a–c). To confirm H2A.Z depletion from chromatin, we conducted ChIP in area CA1 from H2A.Z cKO and control female mice and showed an approximately 25% reduction in H2A.Z binding at *Fos* (t_10_ = 3.23, p = 0.009), *Arc* (t_10_ = 2.53, p = 0.03), *Gadd45b* (t_10_ = 2.29, p = 0.05), and *Fkbp5* (t_9_ = 2.47, p = 0.04) genes, with reduced H2A.Z binding at *Th* approaching significance (t_10_ = 2.05, p = 0.068) (Fig. 2d). Note that H2A.Z deletion was restricted to CaMKIIα-positive cells, and as such, we do not expect a dramatic loss of H2A.Z binding in a mixed cell population. To exclude potential loss of nucleosomes as a source of reduced H2A.Z binding at these loci, we also conducted ChIP against histone H3 and found no changes in H3 binding at any of the above genes (Fig. 2e), suggesting that reduced H2A.Z binding occurs without a loss of nucleosomes.

### H2A.Z has sex-specific effects on fear memory, but not on object-in-place memory

**Figure 3:**
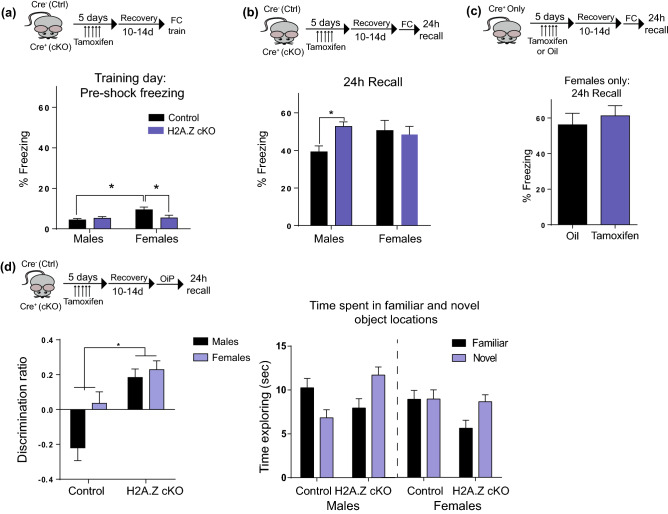
H2A.Z has sex-specific effects on fear memory. (**a**) Control and H2A.Z cKO male and female mice were tested for contextual fear conditioning using a single 0.5 mA foot-shock. Freezing data are shown for (**a**) the training session and (**b**) the 24 h recall test. N = 22–25 for males; N = 12–13/females (**c**) To exclude potential masking effects of tamoxifen on H2A.Z’s function in females, *Cre*^+^ females were treated with either oil or tamoxifen and memory was tested as in *a*. N = 6/group (**d**) A less robust, non aversive form of hippocampus-dependent memory was assessed using the Object in Place (OiP task). Discrimination ratio is shown on the left. Additional analyses of the data show the amount of time spent with objects placed in the familiar *vs* novel location (right), with only H2A.Z cKO mice showing a significant difference between familiar and novel locations, irrespective of genotype. N = 10–13 for males; 15–20 for females. Data are shown as Mean ± SEM, *p ≤ 0.05.

Given that depletion of *H2afz* alone enhanced fear memory in males^34,35^, we tested whether dual deletion of both H2A.Z-encoding genes affects classical fear memory in male and female mice. Analysis of baseline (pre-training) freezing behavior before shock delivery found that control females froze 5 s more on average than control males (p = 0.001), whereas this differences was not seen in H2A.Z cKO mice (Sex × Genotype interaction: F_1,68_ = 5.58, p = 0.02). The lack of a sex difference in H2A.Z cKO mice was driven by reduced freezing in H2A.Z cKO females compared to control females (p = 0.05), which brought H2A.Z-depleted females to the same baseline freezing level as males (Fig. 3a).

To test the effects of H2A.Z cKO on fear memory, freezing behavior was assessed 24 h after a one-shock (0.5 mA) contextual fear conditioning paradigm. Effects of H2A.Z deletion depended both on sex and genotype (Sex × Genotype interaction: F_1,68_ = 4.35, p = 0.04). Consistent with studies of *H2afz* depletion alone, males with dual H2A.Z cKO had enhanced fear memory compared to controls (t_45_ = 3.30, p = 0.02), indicating that the loss of both H2A.Z encoding genes has similar effects on fear memory as loss of *H2afz* alone in male mice. In contrast, H2A.Z deletion had no influence on fear memory in females (Fig. 3b). Given that tamoxifen, which is used to induce Cre activity in H2A.Z cKO mice, acts on the estrogen receptor, we conducted additional experiments to exclude any potential influence of tamoxifen in masking the effects of H2A.Z deletion in females. To this end, additional Cre-positive females were treated with oil (control mice) or tamoxifen (H2A.Z cKO) to control for potential effects of tamoxifen. However, this approach also failed to uncover an effect of H2A.Z cKO on fear memory in females, suggesting that H2A.Z has sex-specific effects on fear memory (Fig. 3c).

Given that fear conditioning is a particularly robust form of learning, we investigated the possibility that a memory-enhancing effect of H2A.Z in females might be revealed under a weaker training paradigm. Object-in-place (OiP) is a particularly challenging memory task in which mice are familiarized with 4 objects that remain the same during training and recall, but 2 out of 4 objects change location during recall, such that the preferential exploration of objects that were moved compared to objects that remain in the same location is taken as an index of memory. All H2A.Z cKO mice performed better than controls irrespective of sex (Main effect of Genotype: F_1,54_ = 20.29, p < 0.001), and females had better overall memory than males (Main effect of Sex: F_1,54_ = 5.11, p = 0.028) (Fig. 3d). We also assessed the time spent exploring novel compared to familiar objects and found a significant interaction between genotype and novelty (F_1,53_ = 20.31, p < 0.0001), whereby H2A.Z cKO, but not control mice, exhibited a significant difference between time spent interacting with objects in familiar *vs* novel locations (t_23_ = 5.78, p < 0.0001), suggesting that only H2A.Z cKO mice exhibited significant learning. H2A.Z deletion improves memory for a non-aversive learning task in both sexes, suggesting that H2A.Z may act as a general memory suppressor in both sexes, but that it has sex-specific effects selectively for fearful stimuli.

### H2A.Z has sex-specific effects on stress-enhanced fear learning (SEFL)

**Figure 4:**
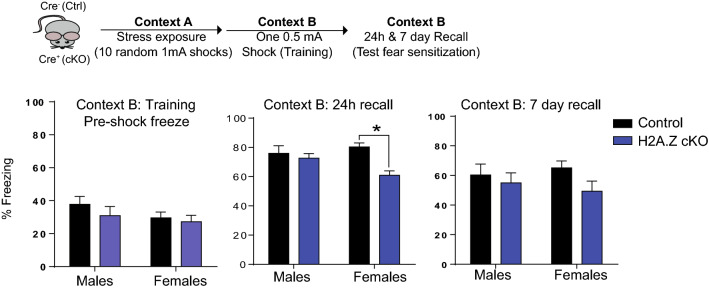
H2A.Z has sex-specific effects on fear memory. Sensitization of new fear learning by a prior stressful experience (an animal model of PTSD) was assessed using the Stress-Enhanced Fear Learning (SEFL) paradigm. The sensitization-inducing stressor consisted of 10 randomly distributed shocks administered over 60 min in Context A. Effects of H2A.Z cKO on fear sensitization were assessed by training mice with a single-shock protocol (single 0.5 mA foot shock) in context B the next day. Data for freezing behavior before shock administration on training day in Context B are shown on the left. H2A.Z cKO females had reduced fear sensitization compared to stress-exposed control mice, as evidenced by reduced freezing 24 h after training in context B. Data are shown as Mean ± SEM, p ≤ 0.05. N = 8–13/group.

Given that H2A.Z selectively impacted fear memory and that there are extensive sex differences in fear-related disorders, such as PTSD, we next investigated whether sex differences in acute fear memory also extend to stress-enhanced fear learning (SEFL), an animal model of PTSD-like symptomatology. SEFL is a model of potentiated fear learning that occurs in PTSD patients and involves trauma-induced potentiation of subsequent fear learning in a novel context^7,9,47,48^. Following a one-hour session with 10 randomly distributed shocks in Context A (mimics traumatic experience), mice were fear conditioned the next day in Context B. We did not find any group differences before shock delivery in Context B (Fig. 4), suggesting that any carry-over effect from receiving shock in context A was not affected by sex or genotype. However, average freezing was ~ 30%, which is higher than the ~ 10% freezing in mice undergoing fear conditioning for the first time (Fig. 3), suggesting that the 1 h SEFL paradigm did produce some degree of generalization to context B. However, this effect did not differ across groups and overall, freezing was ~ 45% lower than it was after training in context B. Specifically, during the 24 h recall test for Context B, H2A.Z cKO females (t_18_ = 4.68, p < 0.0001), but not males, had reduced fear memory compared to controls (Genotype × Sex interaction: F_1,40_ = 4.62, p = 0.04), although this difference was not evident after 7 days (Fig. 4). Thus, even though H2A.Z did not affect acute fear memory in females, it did inhibit potentiation of fear memory by prior stress, an effect that was not observed in males. Moreover, freezing decreased between 24 h and 7 day recall tests irrespective of sex or genotype, indicating that any extinction occurring between days was similar across conditions. Overall, these data suggest that H2A.Z regulates distinct aspects of fear learning in males and females: In males, H2A.Z regulates acute fear learning without altering stress-mediated fear sensitization, whereas the opposite is true in females.

### H2A.Z and SEFL regulate the development of pain hypersensitivity

PTSD patients exhibit altered pain sensitivity compared to the general population^12^, suggesting that sex-specific effects of H2A.Z on fear memory in SEFL-exposed mice may influence pain responses. As with fear memory, pain is subject to sensitization by a variety of noxious stimuli, including an intra-hind paw injection of complete Freund’s adjuvant (CFA), which potentiates sensitivity to thermal and mechanical stimuli for several days^49^. As such, we used the CFA model of chronic inflammatory pain to compare effects of H2A.Z on sensitivity to thermal and mechanical stimulation in mice exposed to SEFL and non-stressed controls.

#### Mechanical withdrawal thresholds

**Figure 5:**
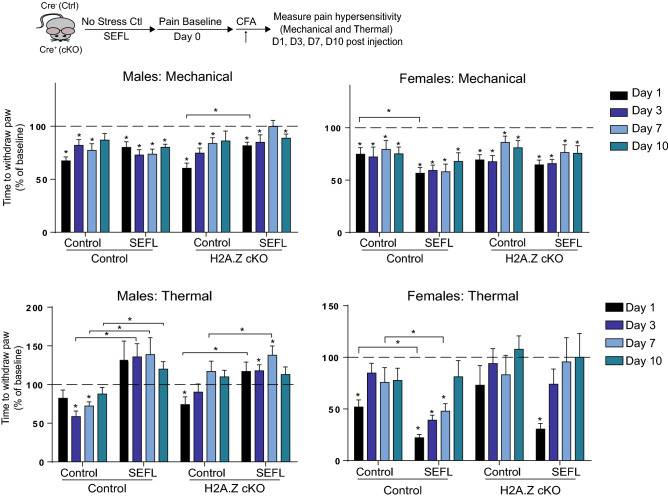
H2A.Z has sex-specific effects on pain hypersensitivity. Given the link between PTSD and pain sensitivity, we tested whether SEFL alters pain hypersensitivity in H2A.Z cKO and control mice using von Frey (mechanical) and radiant heat paw withdrawal (thermal) tests. To test the effect of H2A.Z cKO and SEFL on pain sensitization, mice received a single injection of Complete Frund’s Adjuvant (CFA) into one paw as a model of inflammatory pain, which causes hypersensitivity to mechanical and thermal stimuli. Mice are considered to be hypersensitive when paw withdrawal occurs significantly faster after CFA injection compared to pre-CFA baseline (indicated by the dashed line). Data are shown as Mean ± SEM, p ≤ 0.05. N = 7–13/group.

CFA-induced hypersensitivity and recovery to mechanical stimuli was measured using the von Frey test before and over several days after CFA hind paw administration. In control males without SEFL exposure (Days, Sex × SEFL interaction: F_4,244_ = 3.83, p = 0.005; Days × Genotype interaction: F_4,244_ = 4.01, p = 0.004), CFA treatment reduced withdrawal thresholds to a mechanical stimulus on Days 1, 3, and 7 (all p < 0.05), with recovery occurring by day 10. Control males that were exposed to SEFL had similar response patterns without attaining recovery on day 10 (Day 10: p < 0.0001 *vs* baseline) and SEFL did not influence overall withdrawal threshold on any day (Fig. 5), indicating that SEFL had minimal effect on CFA-induced mechanical hypersensitivity in control males. Similarly, CFA resulted in reduced mechanical withdrawal thresholds in male H2A.Z cKO mice without SEFL exposure on days 1, 3, and 7 (all p < 0.05) and in SEFL exposed H2A.Z cKO mice on days 1, 3, and 10 (p < 0.05). Notably, SEFL-exposed mice had higher mechanical thresholds on day 1 compared to mice without SEFL (t_19_ = 3.69, p = 0.002), suggesting that stress may reduce mechanical pain hypersensitivity in H2A.Z cKO males (Fig. 5).

In females, CFA reduced mechanical thresholds in all mice (control and H2A.Z cKO mice, with and without SEFL) on each of the 4 test days (days 1, 3, and 10: p < 0.05; day 7: p = 0.053 for control without SEFL, p = 0.050 for H2A.Z cKO without SEFL). Additionally, control females exposed to SEFL had lower pain thresholds on day 1 compared to controls without SEFL exposure (t_13_ = 2.16, p = 0.05), suggesting that SEFL enhanced mechanical pain hypersensitivity in females. However, SEFL had no effect in H2A.Z cKO females on any day, suggesting that H2A.Z cKO may protect against SEFL-induced potentiation of mechanical pain (Fig. 5).

#### Thermal withdrawal thresholds

In control males without SEFL exposure (Days × Sex × SEFL interaction: F_3,248_ = 26.89, p < 0.0001; Days × Sex × SEFL × Genotype interaction: F_4,248_ = 2.26, p = 0.06), CFA reduced thermal thresholds on days 3 (t_8_ = 5.79, p < 0.001) and 7 (t_8_ = 5.09, p = 0.001), whereas CFA failed to induce hypersensitivity in mice exposed to SEFL. In fact, SEFL-exposed mice had higher pain thresholds on days 3 (t_17_ = 4.00, p = 0.001), 7 (t_17_ = 2.79, p = 0.01), and 10 (t_17_ = 2.47, p = 0.03) compared to mice without SEFL exposure, suggesting that SEFL inhibits thermal pain hypersensitivity in control males (Fig. 5). Reduced pain sensitivity is paradoxical in light of increased chronic pain in PTSD, but it parallels recent reports from men with PTSD, who show reduced sensitivity to various types of painful stimuli despite higher levels of chronic pain and higher intensity ratings for supra-threshold painful stimuli^12^.

In male H2A.Z cKO mice without SEFL, CFA reduced thermal thresholds only on day 1 (t_7_ = 2.60, p = 0.04) compared to baseline, suggesting rapid recovery from CFA-induced hypersensitivity. In H2A.Z cKO mice exposed to SEFL, CFA increased thermal thresholds on days 3 (t_12_ = 2.28, p = 0.04) and 7 (t_12_ = 3.27, p = 0.008), such that SEFL-exposed H2A.Z cKO males had elevated thermal thresholds on days 1 (t_19_ = 2.46, p = 0.02) and 3 (t_19_ = 2.13, p = 0.046). Thus, SEFL exposure reduced thermal pain sensitivity and this effect was not influenced by H2A.Z in males (Fig. 5).

In control females without SEFL, CFA reduced thermal threshold only on day 1 (t_6_ = 7.1, p < 0.000), whereas SEFL exposure extended this hypersensitivity to days 1 (t_7_ = 24, p < 0.001), 3 (t_7_ = 12.38, p < 0.0001), and 7 (t_7_ = 7.23, p < 0.0001). When compared to control females without SEFL, SEFL reduced thermal thresholds on days 1 (t_13_ = 4.15, p = 0.001) and 3 (t_13_ = 4.47, p = 0.001), suggesting that SEFL potentiated thermal pain hypersensitivity in females, in contrast to reduced hypersensitivity observed in males (Fig. 5). In contrast, H2A.Z cKO females without history of SEFL did not show CFA-induced hypersensitivity, and females exposed to SEFL showed thermal hypersensitivity only on day 1 (t_6_ = 11.06, p < 0.0001). In contrast to control females, H2A.Z cKO females with and without SEFL did not differ from one another on any day, suggesting that H2A.Z deletion is protective against CFA- and SEFL-induced thermal pain hypersensitivity in female mice (Fig. 5).

### Open field and elevated plus maze (EPM)

**Figure 6:**
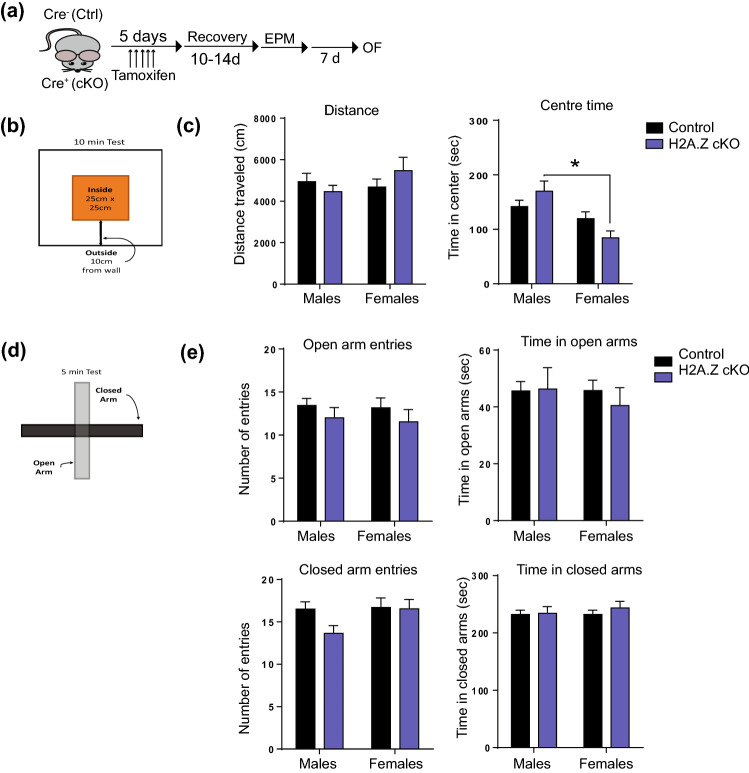
H2A.Z deletion does not impact exploratory or anxiety-like behavior. (**a**) Experimental design. (**b**) Schematic of open-field testing chamber, indicating the area designated as the center. (**c**) Total activity (left) and time spent in the centre (right) for male and female mice. (**d**) Schematic of elevated plus maze. (**e**) Number of entries into the open or closed arm (left) and time spent in the open or closed arms (right). N = 15–16/group for males; 8–12/group for females. Data are shown as Mean ± SEM, p ≤ 0.05.

To exclude potential differences in generalized anxiety as a source of sex and genotype differences in fear memory, additional male and female mice were tested in the open field and the EPM. There were no sex or genotype differences in total distance traveled in the open field, but an analysis of time in the centre identified a significant interaction (F_1,47_ = 4.16, p = 0.047). Although H2A.Z cKO did not influence time in the centre, H2A.Z cKO males spent significantly more time in the centre than H2A.Z cKO females (t_21_ = 3.13, p = 0.005). Neither sex nor genotype influenced any measure of anxiety-like phenotypes in the EPM (time in open and closed arms, number of open or closed arm entries), suggesting that differences in general anxiety are not contributing to differences in fear memory or pain sensitivity (Fig. 6a–e). These data are consistent with evidence that altered pain sensitivity in PTSD patients is not observed in patients with anxiety disorders^12^.

### H2A.Z has sex-specific effects on the expression of plasticity-related genes

**Figure 7:**
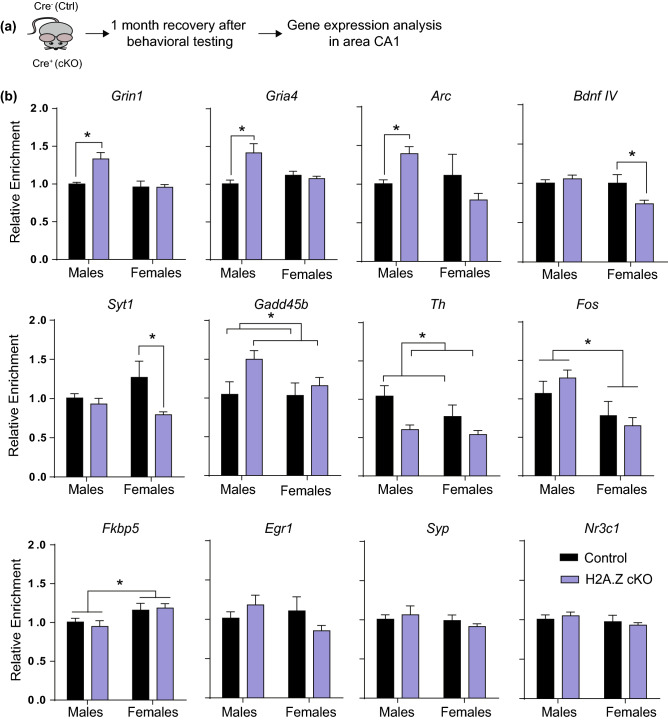
H2A.Z deletion has sex-specific effects on plasticity-related gene expression. (**a**) Brains were collected one month after the completion of behavioral testing (any mice undergoing SEFL were not included). (**b**) H2A.Z cKO produced either sex-specific effects (*Grin1*, *Gria4*, *Arc*, *Bdnf IV*, *Syt1*) or genotype-specific effects (*Gadd45b*, *Th*) on gene expression. Additional genes exhibited sex-specific effects irrespective of H2A.Z cKO (*Fos*, *Fkbp5*), whereas some genes were unaffected by sex or H2A.Z deletion (*Egr1*, *Syp*, *Nr3c1*). N = 4 for control females, 8 for H2A.Z cKO females; 6 for control males, 10 for H2A.Z cKO males. Data are shown as Mean ± SEM, p ≤ 0.05.

To assess the potential gene targets involved in generating distinct behavioral outcomes of H2A.Z in male and female mice, we assessed the effects of conditional H2A.Z deletion on hippocampal expression of several plasticity-related genes (Fig. 7a). Several genes exhibited sex-specific effects of H2A.Z deletion. *Arc* (Sex × Genotype interaction: F_1,23_ = 8.07, p = 0.009), the glutamate receptor *Gria4* (Sex × Genotype interaction: F_1,24_ = 5.23, p = 0.0), and the NMDA subunit *Grin1* (Sex × Genotype interaction: F_1,24_ = 5.22, p = 0.03) showed similar effects, whereby expression was higher in H2A.Z-depleted compared to control males (all p < 0.05) and H2A.Z depletion had no effect in females. In contrast, *Bdnf IV* expression was lower in H2A.Z cKO compared to controls only in females (Sex × Genotype interaction: F_1,24_ = 7.30, p = 0.01). Similarly, only cKO females had reduced expression of the synaptotagmin 1-encoding gene *Syt1* (Sex × Genotype interaction: F_1,24_ = 5.23, p = 0.03) (Fig. 7b).

Several genes were equally affected by H2A.Z cKO in male and female mice, including *Gadd45b* (Main effect of Genotype: F_1,24_ = 4.23, p = 0.05) and the tyrosine-hydroxylase encoding gene *Th* (Main effect of Genotype: F_1,21_ = 11.97, p = 0.002), whereas the expression of other genes varied by sex, but was not affected by H2A.Z. Specifically, the immediate early gene *Fos* (Main effect of Sex: F_1,24_ = 11.17, p = 0.003) was was higher in males than in females. In contrast, *Fkbp5*, a gene implicated in depression and PTSD, was higher in females than in males (Main effect of Sex: F_1,24_ = 7.00, p = 0.01). Other genes were not affected by H2A.Z depletion or sex, including *Egr1*, *B2M*, *Syp* (encodes synaptophysin), and *Nr3c1* (encodes glucocorticoid receptor).

### Estrous cycle

We did not track estrous cycle during any behavioural tasks, even though such factors were previously shown to contribute to cognitive function^50^. To indirectly evaluate the potential contribution of estrous cycle in our behavioral outcomes, we conducted additional analyses to compare the degree of variability within males and within females on each behavioral measure in the manuscript using Levene’s test for the equity of variances. The assumption here was that if estrous cycle is causing a major behavioral effect in females, it would be observed as increased variability in behavioral outcomes within females than within males^50^. No measure resulted in a significant Levene’s statistic (all p > 0.21), thus, we did not observe greater variability in female than in male mice on any of the behaviors reported here, suggesting that estrous cycle was not a major driver of behavior compared to the effect of genotype.

## Discussion

Our data show that hippocampal H2A.Z binding is higher in area CA1 of the hippocampus of females compared to males and that conditional inducible H2A.Z deletion has sex-specific effects on fear memory, SEFL, and hypersensitivity to thermal and mechanical pain stimuli. Importantly, we showed that the depletion of both H2A.Z-encoding genes (*H2afz* and *H2afv*) in CamKIIα-positive cells produces similar memory-enhancing effect in males as does the selective depletion of *H2afz* restricted only to the hippocampus^34,35^, suggesting that H2A.Z depletion in adulthood is a well-tolerated method for improving memory. In contrast to our previous studies that selectively depleted H2A.Z in area CA1 or mPFC^34,35^, the deletion of H2A.Z across brain regions in the current study precludes our ability to attribute our findings to specific brain regions. However, given that effects varied based on the aversive nature of the task and stress history, likely candidates include the hippocampus, amygdala and the mPFC. Although we did not find a similar enhancement of fear memory in females with H2A.Z depletion, both male and female mice exhibited improved memory for the non-aversive object-in-place test, indicating that H2A.Z depletion may provide a strong therapeutic target for improving hippocampus-dependent memory in both sexes.

Notably, sex differences were most prominent in aversive tasks, whereas H2A.Z depletion on the non-aversive OiP task produced improved memory irrespective of sex. This is particularly notable because this task is more difficult than standard object recognition and location tasks, as it includes only three 5-min training phases that include exposure to the same 4 objects used during testing. Indeed, this procedure resulted in particularly weak learning in our control mice, which may be related to the memory-impairing effects of tamoxifen injections administered across conditions^51,52^. Contribution of such factors to subthreshold learning created conditions that reveal a memory-enhancing effect of H2A.Z, consistent with use of subthreshold learning paradigms to reveal enhancing effects of various treatments, including estrogens and histone deacetylase (HDAC) inhibitors^53,54^. As such, these data suggest that H2A.Z depletion may be especially beneficial for reducing the threshold for the formation of non-aversive memories.

H2A.Z had sex-specific effects on SEFL, whereby females with H2A.Z depletion had reduced fear potentiation compared to controls, suggesting that lower levels of H2A.Z may protect against PTSD in females. Interestingly, the protective effect of H2A.Z depletion in females extended to pain sensitivity, whereby H2A.Z cKO females exhibited more rapid recovery from SEFL-induced hypersensitivity to thermal stimuli. This is notable because the beneficial effects of H2A.Z deletion on fear and pain sensitization were not observed in male mice despite similar effects on object-in-place memory, indicating that males and females utilize H2A.Z in different ways when adapting to aversive stimuli.

How differences in behavior relate to higher levels of H2A.Z in females is not clear, but elevated H2A.Z binding in our study is consistent with emerging work on sex differences in the epigenome. Specifically, we previously showed that trimethylation of histone H3 at lysine 4 (H3K4me3) has a high level of overlap with H2A.Z binding^34^, which is consistent with evidence that females have more H3K4me3 binding compared to males^55^. Additionally, DNA methylation, which is inversely related to H2A.Z binding in non-neuronal cells^42,56^ is higher in males than in females^57^. This is particularly relevant because studies in non-neuronal cells showed that artificially elevating DNA methylation results in reduced levels of H2A.Z binding and vice versa^42^, suggesting that sex differences in H2A.Z binding may be associated with lower levels of DNA methylation in females. Although the upstream drivers of this epigenetic shift are not currently known, the sex difference in H2A.Z binding may reflect broader sex difference in the epigenetic landscape. Such differences in the epigenome are consistent with evidence that males and females exhibit widely different transcriptional responses to acute stress within the hippocampus^19^, generating the hypothesis that sex differences in the epigenome help drive unique adaptations to stress in males and females.

Sex differences in molecular adaptations to stress may be especially important for understanding why the effect of H2A.Z on fear memory varied with history of stress, as evidenced by distinct H2A.Z effects to the same fear conditioning procedure when it followed SEFL (Fig. 4) compared to mice without SEFL (Fig. 3). Notably, H2A.Z binding in males can be stably altered after fear conditioning^22^, indicating that experience-induced alterations in H2A.Z may mediate adaptations and responses to subsequent events. Some evidence shows that stress exposure has opposite effects on histone modifications in the frontal cortex and hippocampus of males and females^31^, suggesting that unique adaptations to stress can produce unique responses to subsequent fear-related learning in each sex. With respect to H2A.Z, the shift in its effects under different conditions may reflect its complex role in transcriptional regulation, whereby H2A.Z may facilitate various types of responses in different contexts rather than playing a single and specific role in gene regulation. To this end, H2A.Z has been characterized as a factor that integrates upstream signals to produce variable transcriptional outcomes^58^, consistent with the ability of H2A.Z to recruit both repressive and activating complexes to DNA during development^59^. Moreover, its effect on the activity of associated genes has been linked with the history of the gene’s activity^60^, suggesting that H2A.Z may influence unique responses before and after stress exposure based on interactions with distinct transcriptional regulators under different conditions. Its role may be further influenced by its interactions with sex hormones, which strongly regulate H2A.Z binding in non-neuronal cells and may interact in the same way in the brain to regulate sex-specific outcomes^61^.

Indeed, our gene expression data (Fig. 7) show that the reduction of H2A.Z levels can produce one of 4 gene-specific outcomes on basal transcription in area CA1: (1) an effect on transcription only in males; (2) an effect on transcription only in females; (3) an effect on transcription irrespective of sex; (4) no effect on transcription. This mixed profile is consistent with the view of H2A.Z as a “molecular rheostat” for transcriptional regulation^58^ and its ability to promote the binding of activating and repressive transcriptional regulators to chromatin^59^ to produce varied transcriptional and behavioral outcomes. As such, our behavioral, transcriptional, and H2A.Z-binding data are consistent with a view that H2A.Z integrates diverse upstream signals to facilitate context-relevant outcomes, which appear to vary with history of stress exposure. This contextual specificity of H2A.Z is particularly relevant for the relationship between sex differences in H2A.Z binding (Fig. 1) and sex differences in gene expression produced by H2A.Z depletion on gene expression (Fig. 7). Indeed, there does not appear to be a one-to-one relationship between higher H2A.Z levels in female mice and sex differences in gene expression, indicating that these regulatory differences may emerge specifically in response to particular upstream signals. Moreover, the variable pattern of gene expression with H2A.Z depletion is consistent with our previous reports of gene-specific changes in expression when H2A.Z levels are reduced, whereby some genes are upregulated and others are downregulated in response to H2A.Z depletion^34,35^.

Additionally, we cannot exclude potential interactions of H2A.Z with sex hormones, as reported in non-neuronal cells^62^, as key contributors to sex-specific effects of H2A.Z. This is particularly noteworthy because females in our study were freely cycling and as such, estrous phase may contribute to behavioral outcomes^50^. Although behavioral performance exhibited similar levels of variability in males and females, suggesting minimal contribution of estrous phase compared to the effect of genotype, we did find that certain genes exhibited sex differences in expression irrespective of sex differences in H2A.Z binding. Thus, H2A.Z is a vital contributor to diverse behavioral and transcriptional profiles in males and females, but its role is selective and appears to be modulated by the diverse epigenetic and hormonal landscape that contribute to complex behavioral outcomes. Other examples of sex-specific mechanisms in behavioral regulation have been reported. For example, knockout of glutamate receptor 1 has sex-specific effects on fear memory despite producing similar effects on spatial memory, and epigenetic editing of Cdk5 selectively regulates fear memory in females and not in males^17,63^. Similar effects were recently reported for stress-mediated adaptations to pain, whereby knocking out the nociception/orphanin FQ receptor (NOP) is protective against PTSD-potentiated pain sensitivity in males without having an effect in females^16^. Combined with the results of the present study, these data indicate that stress-induced changes, particularly maladaptive fear and pain sensitization, may be mediated by unique molecular underpinnings in males and females, which has important implications for developing sex-appropriate therapeutic interventions.

This study contributes to growing evidence from our lab for therapeutic potential of H2A.Z inhibition. Specifically, these data expand on our prior findings that selective H2A.Z inhibition in the hippocampus results in enhanced fear memory in males^34,35^ by demonstrating that forebrain-wide reduction of H2A.Z in CaMKIIα-positive cells also improves fear memory in males and OiP memory irrespective of sex. As such, H2A.Z inhibition may be especially beneficial for improving non-aversive memory. However, our data also suggest that potentially beneficial effects of H2A.Z reduction in PTSD and pain treatment may be sex-specific, as benefits of H2A.Z cKO for reducing SEFL-related outcomes were strongest for females. Although there are no H2A.Z-targeting drugs available today, we did demonstrate that pharmacological inhibition of Tip60, which is part of a complex that deposits H2A.Z into chromatin, regulates memory and reduces H2A.Z binding^22^. As such, emerging data are pointing to the need to develop pharmacological strategies that target H2A.Z directly, or indirectly through its upstream regulators.

## Conclusion

Overall, our data extend previous findings that H2A.Z acts as a memory suppressor^22,34,35^ and extend these findings to a non-aversive object-in-place memory task in male and female mice. However, our data show sex-specific effects of H2A.Z on fear memory that depend on history of stress exposure in the SEFL paradigm, indicating that H2A.Z has context-specific effects on regulation of fear memory and related disorders. Specifically, our data suggest that higher levels of H2A.Z in female mice may be a risk factor for PTSD and associated increases in pain sensitivity, and simultaneously point to H2A.Z inhibition as a potential mechanism for reducing risk for these conditions in females. Growing evidence points to unique molecular factors as regulators of risk in males and females, and epigenetic factors may be especially important contributors to this sex differences, as epigenetic changes are evident even when other factors, such as changes in neuronal morphology, are not^64^. H2A.Z in particular may regulate the relationship between stress and pain hypersensitivity, suggesting that sex- and individual differences in H2A.Z regulation may mediate vulnerability to pain in patients with PTSD.

## Methods

### Animals

Conditional-inducible H2A.Z knockout (H2A.Z cKO) mice were generated by crossing H2A.Z.1 (*H2afz*)-floxed and H2A.Z.2 (*H2afv*)-floxed mice (purchased from Riken, RBRC # 05765) with *CamkIIα-CreER*^*T2*^ mice (Jackson Labs Stock # 012362). The H2A.Z cKO were positive for floxed H2A.Z genes and *CamkIIα-CreER*^*T2*^, whereas control mice were positive for floxed H2A.Z genes and negative for *CamkIIα-CreER*^*T2*^. Knockout was induced by tamoxifen injection in adult mice (see below). Male and female mice bred in our colony were group-housed after weaning and were assigned to testing groups at 60–90 days of age. All mice were housed in 7.5 × 11.5 × 5 in cages and maintained on a 12 h light cycle, with ad libitum access to standard mouse chow (Harlan Teklad, Madison, WI) and water. Female mice were freely cycling, and the estrous cycle was not monitored in these experiments. All animal procedures were approved by the University of Toronto Animal Care Committee and performed in accordance with the Canadian Council on Animal Care guidelines.

### Genotyping

Genotyping was carried out at weaning for all mice used in behavioral studies. Animals were ear notched and DNA was extracted using the “HotSHOT” method^65^. The *Cre* transgene was identified by amplifying a unique portion within the *Cre* transgene, as specified by Jackson Labs. Presence of LoxP sites was confirmed by amplifying each target gene (*H2afz* and *H2afv*) with primers specified by Riken (see Table 1), such that the floxed genes resulted in a larger PCR product than wild type genes.

**Table 1:** DNA primers used for genotyping H2A.Z KO mice.

Gene	Forward	Reverse	PCR product size
Jax internal control	5′-CTAGGCCACAGAATTGAAAGATCT	5′-GTAGGTGGAAATTCTAGCATCATCC	324 bp
Cre	5′-AGCTCGTCAATCAAGCTGGT	5′-CAGGTTCTTGCGAACCTCAT	184 bp
H2A.Z1	5′-CGCCTTGGTAATTCTATCTTCTCC	5′-CGCCAGTTAACACACATGTGATC	326 bp (WT) 448 bp (flox)
H2A.Z2	5′-GCCTCAGATCATCCAGTC	5′-GGCTCTGAATTCCCAATGTAG	570 bp (WT) 700 bp (flox)

### Tamoxifen

Tamoxifen (Sigma T5648-5G, final concentration 2 mg/mL) was dissolved in ethanol (40 mg/mL) and mixed with corn oil (375uL tamoxifen in EtOH/750uL corn oil) in 1.5 mL microcentrifuge tubes. After thorough vortexing, ethanol was evaporated by spinning in Eppendorf Vacufuge Plus on V-AL (vacuum-alcohol) setting at 3 °C for 20 min (or until all EtOH had evaporated). Mice received 5 daily injections of tamoxifen (200 mg/kg, IP) beginning at 60–90 days of age and were given 10–14 days to recover before performing additional procedures or testing. All animals (except where specified) were treated with tamoxifen, such that floxed mice without CamkIIα-CreER^T2^ were used as controls. During tamoxifen treatment, mice were housed in ventilated cages (5 × 7 × 14 in.) in a closed-circuit atmosphere rack (Techniplast Easy Flow #BOX110EFUL) and transferred to standard housing thereafter.

### Contextual fear conditioning

To facilitate habituation to the experimenter/non-associative aspects of fear conditioning, mice were transported to the testing room and handled for 30–60 s daily for 3 days before testing, as previously reported^22,34^. On the training and test days, mice were transported to the testing room and placed into test chambers (9.8 in boxes; designed for mice) equipped with an electrified grid floor (Coulbourn Instruments, Holliston, MA, USA). During training, mice were given 2 min to explore the apparatus before receiving a single foot shock (0.5 mA, 2 s), followed by an additional minute of exploration. Memory was assessed 24 h later by replacing the mouse into the training apparatus without shock and measuring freezing behavior for 3 min. Freezing was recorded with a camera placed directly in the chamber ceiling and scored by automated software (FreezeFrame, Coulbourn Instruments).

### Stress-enhanced fear learning (SEFL)

The mouse version of the SEFL paradigm was adapted from Rajbhandari et al. (2018). The chambers and electric shock boxes were purchased from Coulbourn Instruments (Holliston, MA, USA) and the chambers were modified to create 2 distinct training and testing contexts. Context A was differentiated by the following cues: the chamber was wiped down and scented with 1% acetic acid and illuminated by white light. Green plexiglass inserts were placed within the chamber such that the chamber had a green hue and a triangular shape (17.5 cm × 17.5 cm × 23 cm diagonal), and the furniture within the testing room was placed in a triangular format. Context B was illuminated by a red light and cleaned and scented with 5% ammonium hydroxide (Sigma# 221228-500ML-A), the furniture was rearranged along the walls, and no inserts were used, such that the chamber was in an original manufacturer shape (9.8 inch boxes; designed for mice).

Mice were handled for 3 days before testing, which began in Context A. Mice were placed into transfer cages (7.5 × 11.5 × 5 in) and transported on a metal cart to a room (also illuminated with white light) across from the testing room and allowed to habituate for 30 min. They were then placed into individual black wooden boxes (8.5 × 8.5 × 8.5 in.) and transported to the testing room, similar to context A in Rau et al.^9^ to reduce potential generalization between the two contexts. Mice were then placed into the shock chamber and exposed to 10 randomly spaced 1 mA (1 s) shocks over 1 h to mimic a traumatic experience, after which mice returned to home cages.

On day 2, mice were trained with a mild fear conditioning protocol in Context B. Mice remained in their home-cages and were transported on a plastic cart to the hallway outside of the testing room and allowed to habituate for 30 min. Mice were then transported to the testing room two at a time and placed in Context B for 2 min of exploration, followed by a single foot shock (0.5 mA, 2 s), then another minute of exploration. Memory for context B was tested on days 3 and 9 (24 h and 7 day Recall, respectively), wherein mice underwent the same procedures as during the Context B Training day but were exposed to Context B without shock. In all cases, percent freezing was scored by automated software (FreezeFrame, Coubourn Instruments).

### Object in place (OiP)

Mice were transported to the testing room in their home cages and allowed to acclimatize for 30 min. Before behavioral testing, all mice were extensively handled and habituated to an empty testing apparatus (open-field; 45 × 45 × 30 cm) for 10 min on two consecutive days. Mice underwent three 5-min sample phases with 5-min in between each sample. In all sample phases, mice were presented with 4 distinct objects, placed in each corner of the open-field, away from the walls. After a 24 h delay, mice underwent a 5-min choice phase, in which they viewed the same four objects, but two objects had switched locations (right or left, counterbalanced) creating a ‘novel side’. Memory is inferred from the preferential exploration of the objects on the novel compared to the familiar side. The novelty preference was quantified by calculating a discrimination ratio [DR = (novel object exploration – familiar object exploration)/(total object exploration)].

### Pain behavior

#### Complete Freund’s adjuvant (CFA) preparation and administration

Complete Freund’s adjuvant (CFA; 50%; Sigma), used to induce hypersensitivity to pain, was injected subcutaneously in a volume of 20 μl into the left or right plantar hind paw using a 100-μl microsyringe with a 30-gauge needle. Mice were tested for mechanical (von Frey) or thermal (radiant heat withdrawal) sensitivity of both hind paws as described below. Mechanical and thermal testing occurred before, 1, 3, 7 and 10 days post-CFA injection.

#### Von Frey test

Mice were placed into Plexiglas chambers (7 × 6 × 11 cm) on a wire mesh and allowed to acclimate for approximately 1 h. The automated version of this test was used (Ugo Basile Dynamic Plantar Aesthesiometer), whereby a single motor-driven fiber aimed at the animal’s hind paw was applied until the paw was withdrawn. Force was recorded to the nearest 0.1 g. If a cut-off of 20.0 g of force was reached, the probe automatically descended and 20.0 g was recorded as the threshold. Each paw was tested four times with at least 3 min between successive trials, with paw stimulations counterbalanced between animals.

#### Radiant heat paw withdrawal test

Mice were placed into Plexiglas chambers (7 × 6 × 11 cm) on a glass surface and allowed to acclimate for approximately 2 h. The commercial device (IITC model 336) was set to 25% active intensity. Latency was recorded to the nearest 0.01 s. Heating was automatically stopped if the cut-off value of 40.00 s was reached without withdrawal, and 40.00 s was recorded as the latency. Mice were left in the chambers and the contralateral paws were then stimulated. This was repeated four times for each animal, with a minimum of 6 min between same-paw stimulations. Paw stimulations were counterbalanced between mice in an alternating fashion. The mean of the four measurements was considered as the pain threshold for subsequent analyses.

### Elevated plus maze (EPM)

Mice were transported to the testing room in their home cages and were allowed to habituate to for 30 min. They were then placed in the EPM apparatus (arm width 5 cm, arm length from the middle to end: 35 cm, closed arm wall height: 15.25 cm, elevation from floor: 63 cm) facing one of the closed arms and allowed 10 min to explore. Behavior was video recorded by a Sony HD HandyCam (HDR-CX405) and scored manually by an experimenter blind to the experimental conditions and sex of the animals. Number of entries into the closed and open arms, as well as time spent in closed *vs* open arms were scored. Light levels in the open arms were recorded to be 162 lx and in the closed arm 141 lx (Light Meter (LuxMaster 11010067)).

### Open field (OF)

To acclimatize mice to transportation and handling, animals were transported to the behavior testing room in their home cages for 30 min of habituation followed by 30–60 s of handling daily for 3 days before testing. On the test day, mice were transported to the testing room in their home cages and allowed to habituate for 30 min before being placed into the testing chamber (45 cm × 45 cm × 45 cm) for 10 min. Video was recorded using a camera (Microsoft LifeCam Studio) and scored using EthoVision XT 8.5. Time spent in the outer region along the wall (10 cm from the wall) and in the centre (25 cm × 25 cm) was scored using EthoVision XT 8.5 (Noldus Information Technology, Wageningen, The Netherlands). Light levels were recorded to be 10.4 lx at the bottom of the chamber.

### Tissue collection

For RNA and western blot tissue analysis, mice were cervically dislocated and decapitated. Brains were snap frozen in isopentane and stored at − 80 °C until processing, as in previous reports^22,34^.

### Western blotting

Tissues were homogenized using a Dounce homogenizer in RIPA buffer (50 mM Tris HCl pH 7.4, 150 mM NaCl, 10% NP-40, 0.5% sodium deoxycholate, 0.1% SDS) supplemented with Protease Inhibitor Cocktail (Cell Signaling, Danvers, MA, USA), as previously reported^22,34^. Homogenates were incubated for 20 min on ice and flicked every 5 min, centrifuged at maximum speed at 4 °C for 20 min, and the supernatant was collected. Proteins were separated on 8% SDS-PAGE for AR or 15% SDS-PAGE for H2A.Z, then transferred to a PVDF membrane. Membranes were blocked in TBS-T (TBS with 0.1% Tween- 20) containing 5% skim milk and incubated with anti-H2A.Z (C-term) (1:2000, Millipore ABE1348) and anti-Gapdh (1:5,000, Cell Signaling, 5174S, Danvers, MA, USA) antibodies overnight at 4 °C. After three 5 min TBS-T washes, membranes were incubated with HRP secondary antibody (1:10,000 Life Technologies, ThermoFisher, Waltham, MA, USA) for 1 h at RT. Detection was performed by enhanced chemiluminescence and autoradiographs were acquired using ImageQuant LAS 500 (GE) and images were saved as tiff files. Images were not altered between acquisition and analysis of Area Under the Curve using ImageJ (NIH), with H2A.Z normalized to Gapdh from the same membrane.

### mRNA expression and quantitative-PCR (qPCR)

RNA was extracted using BioBasic RNA Extraction Kit (BioBasic #BS82322-250, Amherst, NY), as previously reported^34^. Complementary DNA was synthesized using High Capacity cDNA Reverse Transcription Kit (Applied BioSystems #4368814, Folster, CA, USA). Primers were designed in the lab and used to detect levels of *H2afz* and *H2afv*, which were normalized to the geometric mean of the housekeeping genes *Gapdh* and *β-actin*. A list of primer sequences is provided in Table 2.

**Table 2:** qPCR primers.

Gene	Forward	Reverse
*Actin*	5′-AGATCAAGATCATTGCTCCTCCT	5′-ACGCAGCTCAGTAACAGTCC
*Gapdh*	5′-GTGGAGTCATACTGGAACATGTAG	5′-AATGGTGAAGGTCGGTGTG
*H2afz*	5′-CACCGCAGAGGTACTTGAGTT	5′-TCCTTTCTTCCCGATCAGCG
*H2afv*	5′-CAAGGCTAAGGCGGTGTCTC	5′-CTGCTAACTCCAACACCTCAGC
*Th*	5′-GAGGTATACGCCACGCTGAA	5′-GGAAGCCAGTCCGTTCCTTC
*B2m*	5′-TGCTATCCAGAAAACCCCTCA	5′-TTTCAATGTGAGGCGGGTGG
*Fkbp5*	5′-TGGTGTTCGTTGTTGGGGAA	5′-CCAAAACCATAGCGTGGTCC
*Gadd45b*	5′-CGACAACGCGGTTCAGAAGA	5′-CTGTCGGGGTCCACATTCAT
*Fos*	5′-GGCACTAGAGACGACAGAT	5′-ACAGCCTTTCCTACTACCATTC
*Arc*	5′-CTCTGCTCTTCTTCACTCGGTATG	5′-GAGCTGAAGCCACAAATGC
*Egr1*	5′-GATAACTCGTCTCCACCATCG	5′-AGCGCCTTCAATCCTCAAG
*Syp*	5′-GCCATCTTCGCCTTTGCTAC	5′- CACTTGGTGCAGCCTGAATG
*Bdnf4*	5′-CCAGAGCAGCTGCCTTGATGTTTA	5′-TGCCTTGTCCGTGGACGTTTACTT
*Gria4*	5′-GGACAAGACGAGTGCCTTGA	5′-GCTTCGGAAAAAGTCAGCTTCA
*Grin1*	5′-AAACCTCGACCAACTGTCCT	5′-GTCGTCCTCGCTTGCAGAAA
*Nr3c1*	5′-GGACCACCTCCCAAACTCTG	5′-ATTGTGCTGTCCTTCCACTG

### Chromatin immunoprecipitation (ChIP)

Area CA1 was dissected and samples were incubated in 1% formaldehyde for 10 min at 37 °C, followed by quenching with 1.25 M glycine. Samples were washed with PBS and SDS lysis buffer (50 mM Tris pH 7.4, 10 mM EDTA, 1% SDS, with protease inhibitor cocktail (Cell Signaling Technology) was added to all samples before homogenization and sonication (20 cycles, 30 s on 30 s off, with chromatin shearing beads; bioruptor pico, diagenode). Samples were aliquoted and stored at – 80 °C until use.

Samples were diluted with ChIP dilution buffer (16 mM Tris pH 8, 0.01% SDS, 1% Triton × 100, 1.2 mM EDTA, 167 mM NaCl). 20 µL of Millipore Protein G magnetic beads were added to each sample, with either 1.4 µg of H2A.Z (polyclonal anti-rabbit; Millipore ABE1348) antibody overnight at 4 °C. The next day, samples were washed sequentially with low-salt, high-salt, LiCl (Millipore), and Tris–EDTA (TE; 10 mM Tris, 1 mM EDTA) buffers and rotated for 5 min per wash. Immune complexes from both ChIP and input samples were de-crosslinked and extracted in de-crosslinking buffer (1% SDS, 0.5% Proteinase K, in TE buffer) and heated at 65 °C for 2 h, followed by 95 °C for 10 min before purification with a PCR Purification Kit (Bio Basic). Primers were designed to detect genes of interest (Table 3), and ChIP data were calculated as the percentage of input, then normalized against the control group (DMSO-treated samples).

**Table 3:** Genomic DNA primers used for ChIP-qPCR.

Gene	Forward	Reverse
Fos	5′-AGTGTCTACCCCTGGACCC	5′-GCGTTGAAACCCGAGAACATC
Th	5′-GGGACCTTTCTCTACTACA	5′-CTCTTCCTCAGTTCCCTTA
B2m	5′-GTTCTCCATCCAAAGCTCTA	5′-GCAGAACTCAACCCTTAG
Fkbp5	5′-GCATCTACTTTGCTTACTGA	5′-TGGTTCTCTTCCCTGCTA
Arc	5′-TGCCACACTCGCTAAGCTCC	5′-AACTCCTCTGAGGCAGAAGCC
Gadd45b	5′-CTCTTGGGGATCTTCCGTGG	5′-TCAGCCTCTGTTGTACTCACTT

### Statistics

Analyses were conducted with SPSS Version 24 and consisted of independent-samples t-test when only a single variable was manipulated or two-way ANOVA when multiple variables were manipulated. For pain studies, data were analyzed with mixed measures ANOVA, with Days after CFA injection as the within-subjects factor and Genotype and SEFL as the between-subjects factors. When the omnibus test was significant, follow up analyses were conducted with LSD post-hoc, independent-samples t-test or paired-samples t-test, when appropriate.
